# Neuromuscular blocking agents (NMBA) for COVID-19 acute respiratory distress syndrome: a multicenter observational study

**DOI:** 10.1186/s13054-020-03164-2

**Published:** 2020-07-19

**Authors:** Romain Courcelle, Stéphane Gaudry, Nicolas Serck, Gauthier Blonz, Jean-Baptiste Lascarrou, David Grimaldi, on behalf the COVADIS study group, Nadia Aissaoui, Giuseppe Carbutti, Alain D’hondt, Julien Higny, Geoffrey Horlait, Sami Hraiech, Laurent Lefebvre, Francois Lejeune, Andre Ly, Michael Piagnerelli, Bertrand Sauneuf, Thibaud Soumagne, Piotr Szychowiak, Julien Textoris, Benoit Vandenbunder, Christophe Vinsonneau

**Affiliations:** 1 Unité de soins intensifs, Centres Hospitaliers de Jolimont, La Louvière, Belgium; 2 Réanimation médico-chirurgicale CHU Avicenne, Université Sorbonne Paris Nord, Bobigny, France; 3 Unité de soins intensifs, Clinique Saint Pierre, Ottignies, Belgium; 4 Medecine Intensive Reanimation, District Hospital Center, Boulevard Stephane Moreau, 85000 La Roche Sur Yon, France; 5 Medecine Intensive Reanimation, CHU Nantes, 30 Boulevard Jean Monnet, 44093 Nantes Cedex 9, France; 6 Soins Intensifs, Hôpital Erasme, ULB, Route de Lennik 808, 1070 Bruxelles, Belgium

Dear Editor,

The benefit of neuromuscular blocking agents (NMBA) in acute respiratory distress syndrome (ARDS) is debated [1–3]. Recent guidelines suggest use for most hypoxemic ARDS patients but no more than 48 h [3]. COVID-19 ARDS appears different from classical ARDS [4], and we aimed to describe the use of NMBA and analyze their association with day 28 outcome, in a multicentric observational prospective study (21 ICUs from Belgium and France).

We enrolled consecutive patients with COVID-19 moderate to severe ARDS (Berlin definition) between 10 March and 15 April. The use of NMBA was defined as administration within 24 h after intubation and its duration as the length of continuous paralysis until 24 h of infusion cessation. Further administrations were not considered. Main indications for paralysis were prone positioning and hypoxemia (P/F < 150 mmHg). We dichotomized a priori the NMBA duration in less and more than 2 days. Patients without NMBA were considered to have 0 day of treatment. The primary endpoint was breathing without assistance at day 28. To account for difference between groups, we match the patients with short or long course of NMBA using a propensity score with 0.01 margin. We compared the time to extubation using the Kaplan-Meier curve and log rank test.

Four hundred seven patients with day 28 follow-up were included (Table 1). Among 342 patients (84%) who received NMBA (median duration 5 [IQR 2–10] days), 241 received it for more than 2 days. These latter had higher plateau pressure and rate of prone position and were more frequently in French ICUs. After propensity score matching of 206 patients, the rate and time to breathing without assistance at day 28 did not significantly differ between groups (Table 1 and Fig. 1). This was also the case in the subgroup of more hypoxemic (P/F < 120 mmHg) patients (*n* = 76) and in those with the lowest compliance (< 37 mL/cmH_2_O). Mechanical ventilation tended to be longer in survivors with long NMBA administration.

**Table 1:** Patients’ characteristics and outcome of the study cohort and after propensity score matching

	Study cohort			Propensity score matched		
	≤ 2d ***N*** = 166	> 2d ***N*** = 241	P* value	≤ 2d ***N*** = 103	> 2d ***N*** = 103	P* value
**Medical history**						
**Age, median (IQR)**	**63 (56–71)**	**65 (55–72)**	**0.6**	**62 (56–70)**	**63 (55–72)**	**0.31**
**Gender, male,*n*(%)**	**123 (74)**	**191 (79)**	**0.23**	**71 (68)**	**82 (79)**	**0.11**
**Body mass index, median (IQR)**	**29 (26–32)**	**29 (26–33)**	**0.16**	**29 (27–33)**	**29 (26–32)**	**0.24**
**Chronic pulmonary disease,*n*(%)**	**25 (15)**	**33 (13)**	**0.77**	**14 (0.13)**	**11 (0.10)**	**0.67**
**Charlson score,*n*(%)**						
**0**	**72 (43)**	**98 (41)**	**0.97**	**43 (41)**	**43 (41)**	**0.93**
**1**	**37 (22)**	**66 (27)**		**28 (27)**	**27 (26)**	
**≥ 2**	**57 (34)**	**77 (32)**		**32 (31)**	**33 (32)**	
**Country (France),*n*(%)**	**81 (48)**	**207 (85)**	**< 0.001**	**68 (66)**	**85 (82)**	**0.49**
**Duration of symptoms, days median (IQR)**	**7 (3–9)**	**7 (4–9)**	**0.35**	**7 (5–9)**	**8 (5–10)**	**0.70**
**Respiratory values at baseline**						
**PaO2/FiO2 ratio, median (IQR)**	**126 (88–162)**	**120 (87–157)**	**0.15**	**124 (87–154)**	**120 (81–154)**	**0.47**
**PaO2/FiO2 ratio < 120 mmHg,*n*(%)**	**55 (33)**	**78 (32)**	**0.87**	**37 (35)**	**39 (37)**	**0.77**
**Plateau pressure (cmH**_**2**_**0), median (IQR)**	**23 (20–26)**	**24 (21–26)**	**0.07**	**23 (21–25)**	**24 (21–26)**	**0.9**
**Peep (cmH**_**2**_**0), median (IQR)**	**12 (10–14)**	**11 (10–13)**	**0.38**	**12 (10–14)**	**12 (10–13)**	**0.32**
**Tidal volume, (mL/kg of IBW), median (IQR)**	**6.1 (5.8–6.8)**	**6.1 (5.8–6.6)**	**0.30**	**6.2 (5.8–6.8)**	**6.1 (5.8–6.7)**	**0.26**
**Driving pressure (cmH**_**2**_**0), median (IQR)**	**11 (9–13)**	**12 (10–14)**	**0.07**	**11 (9–13)**	**11 (9–14)**	**0.91**
**Compliance of respiratory system (mL/cmH**_**2**_**O) median IQR**	**36.7 (30.7–44.4)**	**34.6 (27.7–45)**	**0.14**	**36.7 (30.2–40.3)**	**36.8 (28.4–46.7)**	**0.92**
**Prone position,*n*(%)**	**108 (65)**	**217 (90)**	**< 0.001**	**80 (78)**	**82 (80)**	**0.86**
**Outcome**						
**Breathing without assistance at day 28, n (%)**	**81 (49)**	**93 (38)**	**0.04**	**49 (47)**	**45 (43)**	**0.67**
**d14 ventilatory mode**						
**Death**	**41 (25)**	**94 (39)**	**0.001**	**26 (25)**	**35 (34)**	**0.36**
**Controlled mode—ECMO**	**32 (19)**	**55 (23)**		**24 (23)**	**22 (21)**	
**Pressure support**	**50 (30)**	**41 (17)**		**30 (29)**	**21 (20)**	
**Extubated**	**42 (25)**	**51 (21)**		**23 (22)**	**25 (24)**	
**Needs for ECMO,*n*(%)**	**14 (8)**	**31 (13)**	**0.2**	**11 (11)**	**16 (15)**	**0.4**
**d28 VFDs, median (IQR)**	**0 (0–16)**	**0 (0–10)**	**0.005**	**0 (0–15.5)**	**0 (0–12.5)**	**0.25**
**ICU mortality**	**62 (38)**	**98 (41)**	**0.54**	**39 (38)**	**41 (42)**	**0.67**
**Length of MV in ICU survivors, median (IQR)**	**15 (9–26)**	**20 (13–32)**	**0.003**	**15 (9–27)**	**20 (13–31)**	**0.09**
Hospital LOS** in ICU survivors, median (IQR)	**30 (20–50)**	**36 (24–56)**	**0.07**	**31 (20–50)**	**38 (27–52)**	**0.13**

*IQR* interquartile range, *SD* standard deviation, *VFD* ventilatory-free days

*P values were obtained from the Fisher test, *t* test, or Mann-Whitney test as appropriate

**Censured at day 90

**Fig. 1:**
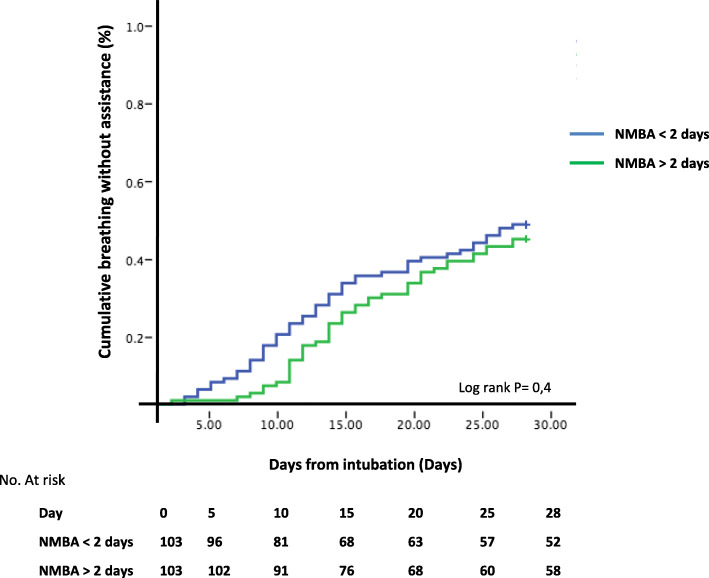
Cumulative proportion of patients breathing without assistance in the two neuromuscular blocker agents (NMBA) groups using Kaplan-Meir curves. Comparison with log rank test

No other secondary outcomes appeared significantly different in this analysis.

In this multicentric study, NMBA were largely used for a longer duration than recommended. In a monocentric study, 60% of 267 mechanically ventilated COVID-19 patients received paralysis [5], whereas in the LUNGSAFE study [6], only 26% of ARDS patients receive NMBA. The massive use of NMBA in the context of shortage during COVID-19 crisis may be questionable. After adjustment for confounders, we did not observe a difference in the proportion of extubation rate according to NMBA length.

The high compliance of COVID-19 ARDS should protect them from barotrauma [4], one of the protective effects of NMBA [2]. However, respiratory drive appears high in these patients and patient self-induced lung injury (P-SILI) may occur, which may explain why investigators administered NMBA. We observed a low plateau pressure in our study but have no data about potential P-SILI.

One can be surprised to observe that matched on severity, time to extubation at day 28 was similar between patients with short or long course of NMBA. Our results indicate an equipoise regarding the duration of NMBA, which should be tested in proper trials.

Our study has limitations. Confounding and indication biases may exist despite adjustment, which decreased the study power. We did not collect the reason for NMBA continuing; they may have been prolonged in patients with the worst evolution. Finally, we did not gather the use of light sedation [1] and the occurrence of ICU-acquired weakness and diaphragm paresis, suggested by the trend to higher length of ventilation in the survivors.

In conclusion, we observed a large and prolonged use of NMBA in COVID-19 ARDS.

After adjustment, a prolonged course of NMBA was not associated with a lower rate of extubation at day 28.

## Data Availability

D. Grimaldi and JB. Lascarrou had full access to all the data in the study and had final responsibility for the decision to submit for publication. The database will be public within 3 months after publication at https://icucovadis.com
